# Molecular game theory for a toxin-dominant food chain model

**DOI:** 10.1093/nsr/nwz097

**Published:** 2019-07-19

**Authors:** Bowen Li, Jonathan R Silva, Xiancui Lu, Lei Luo, Yunfei Wang, Lizhen Xu, Aerziguli Aierken, Zhanserik Shynykul, Peter Muiruri Kamau, Anna Luo, Jian Yang, Deyuan Su, Fan Yang, Jianmin Cui, Shilong Yang, Ren Lai

**Affiliations:** 1 Key Laboratory of Animal Models and Human Disease Mechanisms of Chinese Academy of Sciences/Key Laboratory of Bioactive Peptides of Yunnan Province, Kunming Institute of Zoology, Kunming 650223, China; 2 Department of Biomedical Engineering, Washington University St. Louis, MO 63130, UK; 3 Key Laboratory of Medical Neurobiology, Department of Biophysics and Kidney Disease Center, First Affiliated Hospital, Institute of Neuroscience, National Health Commission and Chinese Academy of Medical Sciences, Zhejiang University School of Medicine, Hangzhou 310058, China; 4 Department of Biological Sciences, Columbia University, New York, NY 10027, UK; 5 KIZ/CUHK Joint Laboratory of Bioresources and Molecular Research in Common Diseases, Kunming 650223, China; 6 University of Chinese Academy of Sciences, Beijing 100049, China

**Keywords:** toxin, receptor, molecular game, amphibian, scorpion

## Abstract

Animal toxins that are used to subdue prey and deter predators act as the key drivers in natural food chains and ecosystems. However, the predators of venomous animals may exploit feeding adaptation strategies to overcome toxins their prey produce. Much remains unknown about the genetic and molecular game process in the toxin-dominant food chain model. Here, we show an evolutionary strategy in different trophic levels of scorpion-eating amphibians, scorpions and insects, representing each predation relationship in habitats dominated by the paralytic toxins of scorpions. For scorpions preying on insects, we found that the scorpion α-toxins irreversibly activate the skeletal muscle sodium channel of their prey (insect, BgNa_V_1) through a membrane delivery mechanism and an efficient binding with the Asp/Lys-Tyr motif of BgNa_V_1. However, in the predatory game between frogs and scorpions, with a single point mutation (Lys to Glu) in this motif of the frog's skeletal muscle sodium channel (fNa_V_1.4), fNa_V_1.4 breaks this interaction and diminishes muscular toxicity to the frog; thus, frogs can regularly prey on scorpions without showing paralysis. Interestingly, this molecular strategy also has been employed by some other scorpion-eating amphibians, especially anurans. In contrast to these amphibians, the Asp/Lys-Tyr motifs are structurally and functionally conserved in other animals that do not prey on scorpions. Together, our findings elucidate the protein-protein interacting mechanism of a toxin-dominant predator-prey system, implying the evolutionary game theory at a molecular level.

## INTRODUCTION

Evolution has fine-tuned the ability of venoms in many venomous animals, such as snakes, spiders, centipedes and scorpions, to rapidly incapacitate both prey and predators—especially for fast-moving targets—as a mechanism for hunting prey or deterring predators [1–4]. To achieve a paralytic envenomation, targeting skeletal muscle sodium channel Na_V_1.4 in mammals or its counterpart receptor BgNa_V_1 in insects is an efficient strategy, because this channel is crucial for skeletal muscle contraction as it regulates the generation and propagation of action potentials [5–8]. During the long evolutionary game process of natural selection, the formation of food chains containing venomous animals was likely dominated by venom components that elicited muscular toxicity [9,10].

Scorpions (*Mesobuthus martensii* Karsch) inflict potentially paralytic and lethal stings mainly through their α-toxins acting on the skeletal muscle sodium channel [11–13]. Scorpion α-toxins slow or inhibit the inactivation process of Na_V_ channels and thus induce prolongation of action potentials [14–16]. For scorpions, causing the dysfunction of the muscular system by α-toxins is a unique evolutionary and molecular mechanism that rapidly renders prey incapable of retaliation or escape. Although toxins have been underlined by their powerful bioactivities and were thought to be crucial for predation, venomous animals are not located at the top of the food chain in most ecosystems [17–19]. This raises questions regarding how the higher players maintain their dominance in the food chain and how they invalidate their preys' toxins.

**Figure 1.**
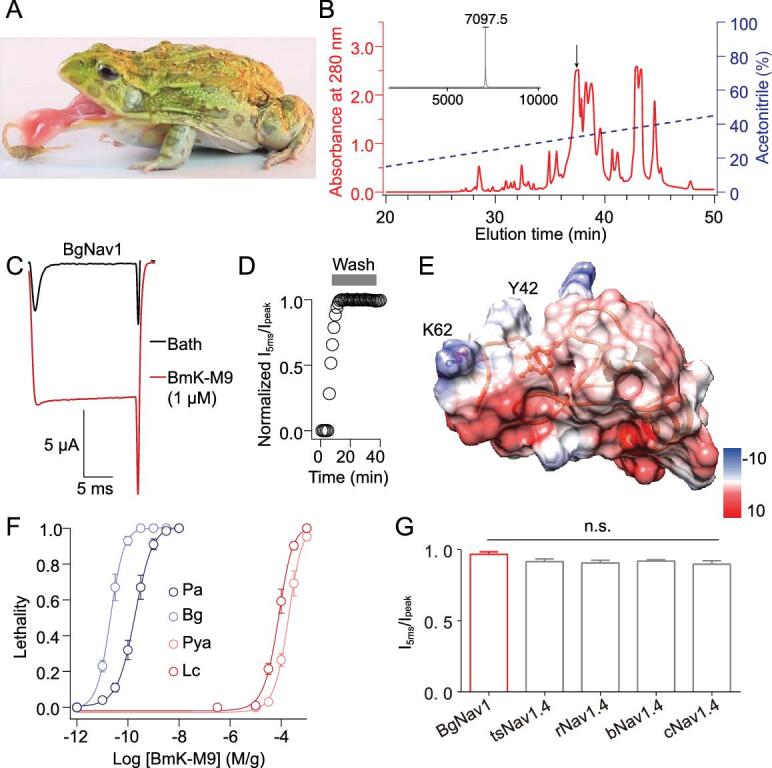
Frogs exhibit significant tolerance to α-toxins. (A) Image of a frog (*Pyxicephalus adspersus*) preying on a scorpion (*Mesobuthus martensii)*. (B) Isolation of native BmK-M9 (blue arrow) from the pooled protein fractions by a C_18_ RP-HPLC column. (Inset) The purity and molecular weight of BmK-M9 were identified by MALDI-TOF analysis. (C) Representative BgNa_V_1 currents from a two-electrode voltage-clamp recording in the presence of 10 μM BmK-M9. (D) Association and dissociation analysis of the interaction between BmK-M9 and BgNa_V_1. The association trace was plotted by applying 10 μM BmK-M9 and dissociation trace was plotted by washing with bath solution within 30 minutes. The oocytes were perfused by 10 μM BmK-M9 for 30 seconds to ensure the saturated concentration of the toxin on the channel. (E) The structural model of BmK-M9 with the electrostatic potential distribution shown in color (red = negative, blue = positive) on the right. The location of two key residues Y42 and K62 are shown on the BmK-M9 structure. (F) A dose-response curve for the lethal effect of BmK-M9 determined 24 hours after injecting into two kinds of cockroaches (*Blattella germanica*, Bg, and *Periplaneta americana*, Pa) and two kinds of frogs (*Pyxicephalus adspersus*, Pya, and *Lithobates catesbeianus*, Lc). Data points were fitted according to the Hill equation. Average values are given as mean ± SEM; n = 30 cockroaches per group and n = 5 frogs per group. n = 3 groups per data point. (G) Comparison of the I_5 ms_/I_peak_ values of BmK-M9 treated Na_V_1.4 channels of cockroach (BgNa_V_1), tree shrew (tsNa_V_1.4), rat (rNa_V_1.4), bat (bNa_V_1.4) and camel (cNa_V_1.4).

Interestingly, as predators of scorpions, some amphibians might have evolved a tolerance to the assault of scorpion toxins on the muscular system [20]. Anuran species, such as *Leptodactylus pentadactylus*, *plethodantohyla inguinalis* [21,22], have been recorded to prey on scorpions and this evolutionary phenomenon is supported by several physiological mechanisms at a molecular level. Based on these observations, we questioned whether the frog or toad employs certain resistance mechanisms to the paralytic toxins of scorpion and whether these molecular strategies are crucial for this predator-prey relationship. Our observations showed that the frog (*Pyxicephalus adspersus*) exhibited resistance to stings of the scorpion (*Mesobuthus martensii*), preyed and consumed the scorpion without a paralytic response (Supplementary Movie S1, available as Supplementary Data at *NSR* online). This suggests that the frog evolved to reduce sensitivity to the paralytic toxins. By contrast, these toxic stings play an extremely successful defensive or lethal role in other vertebrates and insects [23–25]. Are scorpion α-toxins invalidated by encountering a detoxification mechanism in frogs? In the present study, we unraveled the molecular strategies and the evolutionary game theory in a food cycle composed of anuran species, scorpions and insects through integrating the results from animal tests, electrophysiology, mutagenesis, fluorescent dynamics and computational modeling.

**Figure 2.**
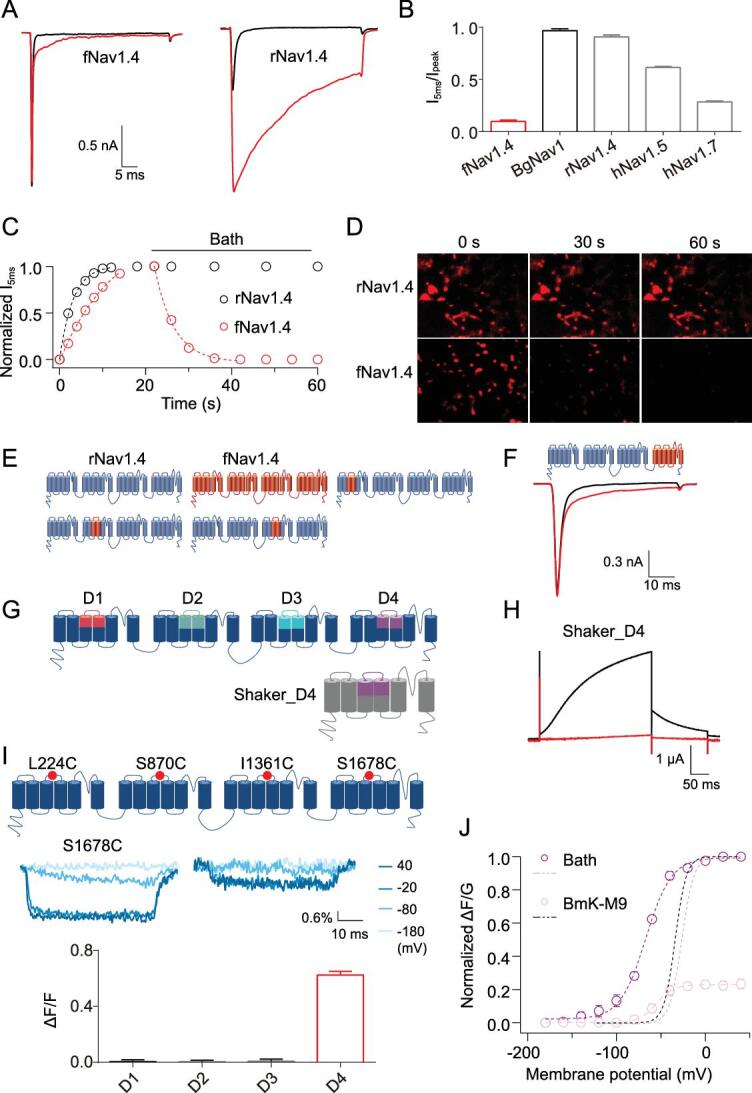
DIV of fNa_V_1.4 possesses the resistance property to α-toxins. (A) Representative whole-cell currents of fNa_V_1.4 and rNa_V_1.4 before and after 10 μM BmK-M9 application. The cells were perfused by 10 μM BmK-M9 for 30 seconds to ensure a saturated concentration of the toxin on the channel. (B) The I_5 ms_/I_peak_ value of each sodium channel following application of 10 μM BmK-M9. The statistical values are given as mean ± SEM (n = 3 cells). (C) Comparison of binding affinity of BmK-M9 on rNa_V_1.4 and fNa_V_1.4. The association traces were plotted by application of 10 μM BmK-M9 and dissociation traces were plotted by washing with bath solution within 40 seconds. (D) The toxin dissociation rate was recorded following a series of images from rNa_V_1.4-expressing (top row) and fNa_V_1.4-expressing (bottom row) HEK293T cells incubated with F-BmK-M9 (10 μM). (E) Schematic representation of the chimeras between rNa_V_1.4 (blue) and fNa_V_1.4 (red). (F) Representative whole-cell currents of DIV chimeric channel before and after 10 μM BmK-M9 application. (G) Schematic representation of the chimeras between BgNa_V_1 (blue) and Shaker (grey). (H) Representative whole-cell currents of Shaker_D4 chimeric channel were recorded before and after 10 μM BmK-M9 application. (I) Diagram of BgNa_V_1 channels indicating the location of mutated cysteine labeled with TAMRA-MTS (up); Fluorescence signals evoked at −180 mV, −80 mV, −20 mV, 40 mV were recorded from channel mutant S1678C before (left panel) and after (right panel) 10 μM BmK-M9 application (middle); The changes in fluorescence signals (at 40 mV) for the four fluorescence-labeled channel mutants were analysed in the presence of 10 μM BmK-M9. The statistical values are given as mean ± SEM (n = 3 cells) (down). (J) The voltage-dependent fluorescence (circle) and conductance-voltage relationship (dash line) of channel mutant S1678C were analysed before and after 10 μM BmK-M9 application. The statistical values are given as mean ± SEM (n = 3 cells).

## RESULTS

### Significant tolerance of frog-to-scorpion stings and α-toxin

As shown in Fig. 1A and Supplementary Movie S1 (available as Supplementary Data at *NSR* online), although the scorpion displayed a powerful chemical defense via several toxic stings, these stings had no impact on the frog during the predatory process. By contrast, scorpion stings exhibited a successful defensive role in laboratory mice (Supplementary Movie S2, available as Supplementary Data at *NSR* online). Therefore, frogs may possess some molecular strategies to diminish the physiological effects induced by these toxins. To obtain a representative muscle-paralytic α-toxin, considered as a major component for subduing prey and deterring predators, we purified an α-toxin (BmK-M9) in abundance (Fig. 1B and Supplementary Fig. S1A, available as Supplementary Data at *NSR* online) [26] from the crude venom of the scorpion (*Mesobuthus martensii*). Functional tests indicated a potent bioactivity of BmK-M9 on the insect muscular sodium channel (BgNa_V_1), with complete elimination of fast inactivation of the channel (Fig. 1C). The interaction between BmK-M9 and BgNa_V_1 is distinctly strong, yielding an extremely slow dissociation (Fig. 1D). Due to the high affinity and bioactivity of BmK-M9 on insects among known α-toxins (Supplementary Table S1 and Fig. S1B–E, available as Supplementary Data at *NSR* online), we used this toxin as a prototypic α-toxin and established the homologous model of BmK-M9 (Fig. 1E). As expected, by calculating the lethal dosage, we found that cockroaches exhibited more than 100,000-fold sensitivity to BmK-M9 compared to frogs (Fig. 1F). Given that the skeletal muscle sodium channels are the general main target of scorpion α-toxins (Fig. 1G) [12,13,27–29], we hypothesized that the counterpart receptor (frog Na_V_1.4, fNa_V_1.4) of the BgNa_V_1 channel may be the molecular basis of frog's detoxification mechanism, thus making the frog a higher-level predator in this food chain.

### Domain IV of fNa_V_1.4 and BgNa_V_1 determines the sensitivity to α-toxin

To test our hypothesis, we first cloned fNa_V_1.4 from a frog (*Pyxicephalus adspersus*) and expressed it in HEK293 cells. As shown in Supplementary Fig. S2A (available as Supplementary Data at *NSR* online), the steady activation of fNa_V_1.4 (V_a1/2_ = −28.1 mV) is similar to that of the BgNa_V_1 channel (V_a1/2_ = −26.5 mV). By comparing the fraction of remaining current at 5 milliseconds after the peak versus the peak current amplitude (Table S2, available as Supplementary Data at *NSR* online), fNa_V_1.4 possesses unique molecular mechanisms that significantly resist BmK-M9, unlike BgNa_V_1 (Fig. 2A–C). In agreement with our electrophysiological results, fluorophore-linked BmK-M9 (F-BmK-M9) revealed the fast dissociation of BmK-M9 on toxin-insensitive fNa_V_1.4-expressing cells (Fig. 2D). To focus on the structural basis of fNa_V_1.4 containing the resistance, a series of chimeric channels were made between fNa_V_1.4 and mammalian Na_V_1.4 (Fig. 2E and F). We found that only the homologous domain IV (DIV) of fNa_V_1.4 retained its resistance properties to BmK-M9 (Fig. 2F and Supplementary Fig. S2B, (available as Supplementary Data at *NSR* online). A previously reported approach [30] helped us to further confirm the interaction between BmK-M9 and the voltage-sensing domains (VSDs), in which specific VSD paddles from each homologous domain of BgNa_V_1 channel were transplanted into a Shaker channel (Fig. 2G). Consistently, 1 μM BmK-M9 exclusively interacted with the DIV-VSD construct (Shaker_D4), whereas domain I, II, III constructs and WT Shaker were unaffected (Fig. 2H, Supplementary Fig. S2C and D, available as Supplementary Data at *NSR* online). Saturated BmK-M9 partially inhibited the gating current of BgNa_V_1 and completely suppressed that of Shaker_D4 (Supplementary Fig. S3A and B, available as Supplementary Data at *NSR* online). We also labeled a fluorophore (TAMRA-MTS) onto four VSDs (L224C, S870C, I1361C and S1678C, Fig. 2I, Supplementary Fig. S3C and D, available as Supplementary Data at *NSR* online) of BgNa_V_1 to track their movements during the channel-gating process [31–33]. Similarly, 1 μM BmK-M9 notably decreased the fluorescence signals of S1678C labeled channel mutants and shifted the fluorescence curve to depolarized potentials by about 25 mV (Fig. 2I and J). These results together suggest that BmK-M9 occludes the movement of insect or mammal-derived DIV-VSD, which is expected not to occur in fNa_V_1.4.

**Figure 3.**
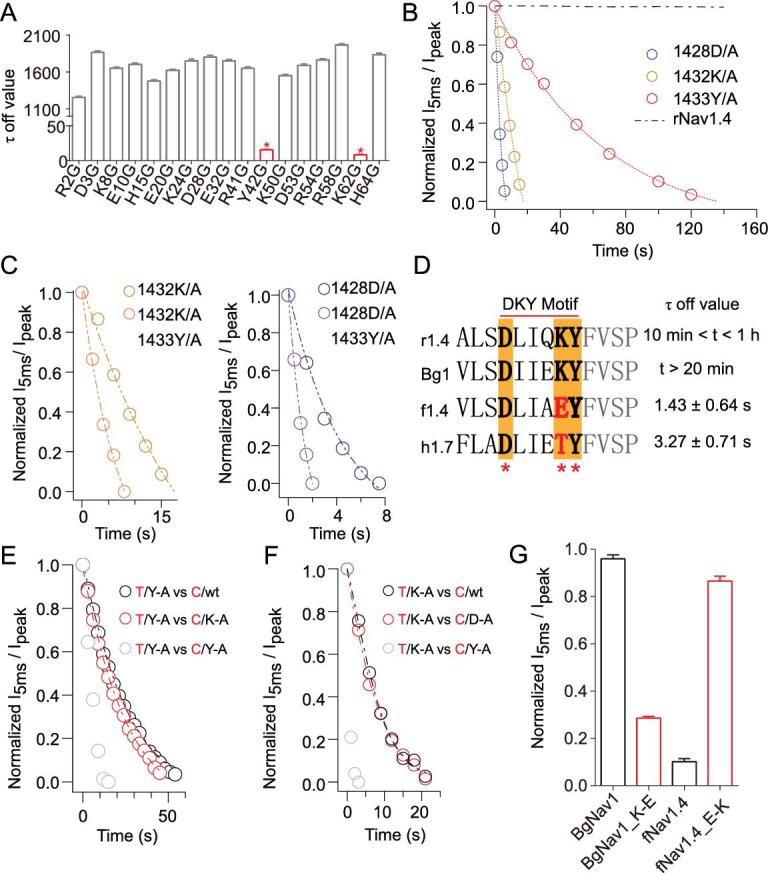
A glutamic acid mutation in the Asp/Lys-Tyr motif provides the species selectivity for α-toxins. (A) Screening τ_off_ values of 17 single-point toxin mutants on rNa_V_1.4. (B) Dissociation of BmK-M9 on rNa_V_1.4 and three single-point channel mutants. (C) Comparison of the dissociation traces of BmK-M9 on single-point mutant K1432A and double-point mutant K1432A/Y1433A (left); single point-mutant D1428A and double-point mutant D1428A/Y1433A channel (right). (D) Sequence alignment of the Asp/Lys-Tyr motif of rNa_V_1.4 (r1.4), BgNa_V_1 (Bg1), fNa_V_1.4 (f1.4) and hNa_V_1.7 (h1.7). The corresponding τ_off_ values of BmK-M9 on these channels are shown. (E, F) The dissociation traces of toxin (T in red) mutants on channel (C in red) mutants. (G) Comparison of the I_5 ms_/I_peak_ values on BmK-M9 treated BgNa_V_1 and fNa_V_1.4 with a single amino acid change.

### A point mutation bestows fNa_V_1.4 with resistance to paralytic α-toxin

Additionally, glycine/alanine screening revealed the key residues in the toxin-channel interaction. Two residues (42Y and 62K) were identified as the key sites of the toxin by the washing-out time-course analysis, yielding τ-off values of 25.82 and 15.07 seconds, respectively (Fig. 3A). Asp/Lys-Tyr motif was identified as the binding pocket of BmK-M9, given that the three residues located in this motif of mammalian DIV-VSD were found to be important for the toxin-channel interaction (Fig. 3B–D). To experimentally test the site-to-site interaction, we employed an analysis based on thermodynamic mutant cycling [34,35]. Briefly, if one of these two residues specifically interacts with one residue in the Asp/Lys-Tyr motif, then the τ-off value of double mutation should be nonadditive compared with that of a single mutation. Otherwise, the effects of accelerating the decrease in I_5ms_/I_max_ value by mutating these residues would be additive. Except for fNa_V_1.4, the tyrosine (42Y) of BmK-M9 directly interacts with the lysine located in Asp/Lys-Tyr motif (Fig. 3E and F), which likely provides the species selectivity for BmK-M9 (Supplementary Fig. S4A, available as Supplementary Data at *NSR* online). Based on these understandings of the site-to-site interaction, it is hardly surprising that a single-point mutation could largely alter the bioactivity of BmK-M9 on both fNa_V_1.4 and mammalian Na_V_1.4 (Fig. 3G). Compared to the skeletal muscle sodium channels of other animals, a glutamic acidin the Asp/Lys-Tyr motif of fNa_V_1.4 acts as the molecular determinant and reverses the charge by replacing lysine in this motif, which may bestow the frog with biological resistance to the paralytic α-toxin.

**Figure 4.**
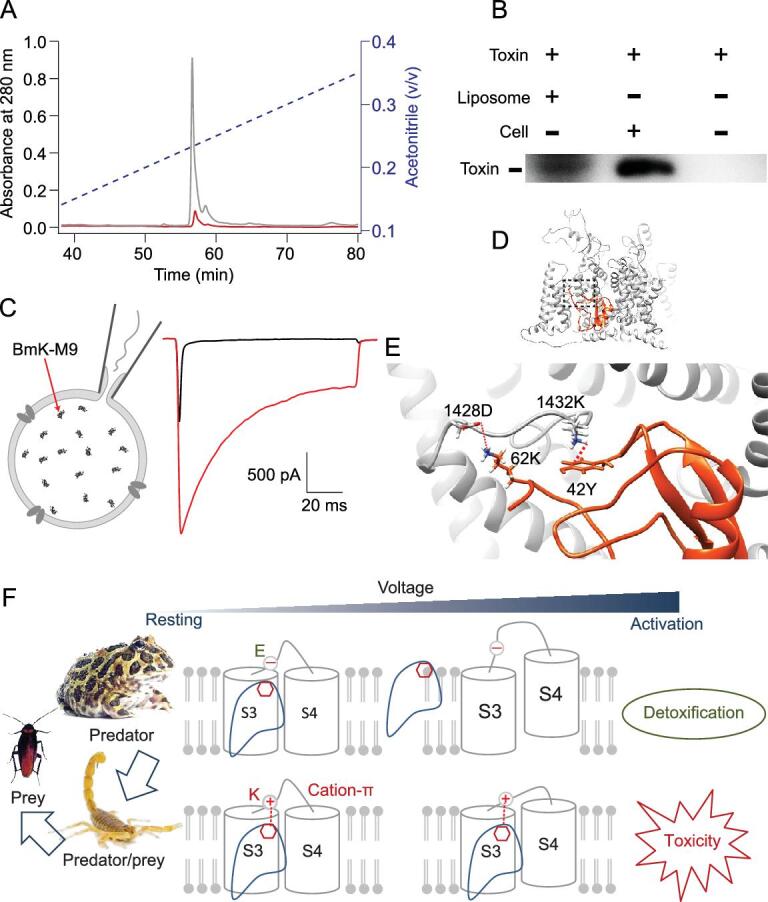
The detoxification receptor fNa_V_1.4 repels the binding of α-toxins. (A) The RP-HPLC detection of BmK-M9 in DMEM medium supernatant with HEK293T cells (red) and without (grey) HEK293T cells. BmK-M9 was detected after 24 hours of toxin application. (B) The interaction between BmK-M9 and lipid membranes. The bands represent the toxin extracted from liposome and lipid-bilayer membranes of HEK293T cells, which were incubated with 10 μM BmK-M9, respectively. The 10 μM BmK-M9 in DMEM medium without lipid membranes was used as control. (C) Representative whole-cell currents of rNa_V_1.4 at the beginning of whole-cell formation and 30 seconds after whole-cell patch constructed whereby 10 μM BmK-M9 was added in the pipette solution. (D) The docking model of BmK-M9 with domain IV of rNa_V_1.4 at resting state. (E) A zoomed-in view of the interaction between BmK-M9 and rNa_V_1.4. (F) A cartoon showing the molecular game theory for a toxin-dominant food chain model among frogs, scorpions and insects.

### Structural and molecular strategies of the predatory game process

We used Rosetta to simulate the resting state of DIV-VSD by aligning the first arginine (R1448) resolved in the activated state (PDB: 6AGF) to the fourth arginine (R1457) and rebuild the loop between transmembrane segment 3 and 4. As shown in Supplementary Fig. S4B (available as Supplementary Data at *NSR* online), the Asp/Lys-Tyr motif is embedded in the lipid membrane in the resting state model, suggesting a lipid-dependent interaction between toxin and DIV-VSD. BmK-M9 was incorporated into the cell membrane in our partition experiments (Fig. 4A and B). In agreement with the toxin-membrane interaction, BmK-M9 showed effective bioactivity when applied from the intracellular side (Fig. 4C). When BmK-M9 was docked into this resting state model, the toxin largely resided within the membrane (Fig. 4D). The docking model is fully consistent with our results from point-mutation screening, toxin-lipid partition experiments and patch-clamp recordings. In the zoomed-in complex model, a cation-π interaction between BmK-M9 and the Asp/Lys-Tyr motif of BgNa_V_1 is necessary to stabilize the location of DIV-VSD in its resting state (Fig. 4E). Given that this glutamic acid mutation could be observed in the skeletal muscle sodium channel of sequenced anuran species (Supplementary Fig. S4A, available as Supplementary Data at *NSR* online), it likely provides a detoxification mechanism for scorpion-eating amphibians, especially anurans. Therefore, this mechanism minimizes muscular toxicity induced by scorpion stings, thus, frogs prey and ingest them without paralytic responses (Fig. 4F).

## DISCUSSION

Venomous animals are consistently excellent predators due to possession of formidable venom biochemical armaments and thereby often occupy dominant positions in food chains [36,37]. To be higher-level predators of these venomous animals, evolutionary game processes at the molecular level are necessary to equip several crucial detoxification mechanisms that circumvent the risk of poisoning [38,39]. The present study highlights a representative example of this type of predatory game theory among scorpion-eating amphibians, scorpions and insects.

Scorpions evolved a gene-encoded venom system as a primary chemical weapon for capturing prey [40,41]. Recent cryo-EM studies have resolved the atomic structures of several toxin-Na_V_ channel complexes, showing that toxins engage with the channel mainly through protein-protein interactions [42–45]. However, we find that to tightly fix DIV-VSD in the resting configuration, scorpion α-toxin not only interacts with this domain through a salt bridge and a cation-π interaction, but it also penetrates deeply into the lipid membrane and holds DIV-VSD against activation (Fig. 4D–E). These characteristics of α-toxins make the prey's skeletal muscle sodium channels constitutively activated without any inactivation, resulting in muscle rigidity. We found the important role of DIV-VSD (Fig. 2E–J), especially the Asp/Lys-Tyr motif (Fig. 3B–D), which is embedded in the cell membrane at its resting state (Fig. 4A–E, Supplementary Fig. S4B, available as Supplementary Data at *NSR* online). Therefore, paralytic α-toxin obviously gained an upper hand in the predatory game between scorpions and their prey.

Scorpion stings containing these paralytic α-toxins also exhibit defensive roles against vertebrates, like rodents (Supplementary Movie S2, available as Supplementary Data at *NSR* online), by exploiting the same mechanisms. This poses, however, a different question of why rodents do not evolve tolerance. We first rule out the possibility that the rodents' attack is swift enough to ingest scorpions directly and avoid scorpion stings (Supplementary Movie S2, available as Supplementary Data at *NSR* online), because scorpion sting events (in mini-second range) are too fast to dodge [46,47]. It is plausible that rodents have no need to exploit an otherwise new toxic food source and also do not get stung by scorpion frequently, given that they are not in the same food cycle. Supporting this argument, the projection behavior of frogs' tongues may reduce the number of scorpion sting and envenomation capacity during the predation process. Moreover, at a molecular level, resistance to scorpion stings, especially in frogs, is more likely a receptor-benefit result of a long-term prey and predation game. In fNa_V_1.4, the Asp/Lys-Tyr motif is mutated to the Asp/Glu-Tyr, one of attracting pair forces that mutually repel, which greatly reduces the affinity of the toxin for fNa_V_1.4 (Fig. 4F). Except for scorpion-eating amphibians, such as the anuran species, we found that the Asp/Lys-Tyr motif is conserved in nonscorpion-eating vertebrates. Interestingly, the Asp/Lys-Tyr motif of nonscorpion-eating amphibians, such as salamanders, also is intact (Supplementary Fig. S4A, available as Supplementary Data at *NSR* online). Our results provide a clue that specie-crossing interactions hide more delicate molecular mechanisms among ligands and receptors to support the interesting and intense coevolutionary game.

## MATERIALS AND METHODS

### Ethics statement

All of the animal experiments were performed in accordance with recommendations in the Guide for the Care and Use of Laboratory Animals of Kunming Institute of Zoology, Chinese Academy of Sciences. Experimental protocols using animals in this study were approved by the Institutional Animal Care and Use Committees at Kunming Institute of Zoology, Chinese Academy of Sciences (approval ID: SMKX-2018029).

### Purification and protein sequencing of BmK-M9

A total of 1,000 (both sexes) adults *Mesobuthus martensii* were purchased from Shandong Province, China. As previously reported [48], crude venom was collected by stimulating the venom glands with a 3 V alternating current. BmK-M9 was purified from the crude venom by using a combination of a Sephadex G-50 gel filtration column and reverse-phase (RP)-HPLC. The purity and molecular weight of the toxin were analysed using a matrix-assisted laser desorption ionization time-of-flight (MALDI-TOF). The toxin with a purity of over 99.8% was collected and stored at −80°C. A Shimadzu protein sequencer (PPSQ-31A, Shimadzu, Japan) was used for the determination of the amino acid sequence of BmK-M9.

### Preparation of recombinant toxin and fluorescent-labeled BmK-M9

Expression vector construction, protein expression and purification were performed as described previously [49,50] with fine tuning. In summary, the cDNA encoding BmK-M9 was synthesized with codons optimized for expression in *Escherichia coli*, and it was cloned into the modified expression vector pet32a (+) (Novagen). This vector (pet32a) encodes a His6 tag for affinity purification, a Trx-Tag for improving the solubility and activity of the expressed peptide, and a tobacco etch virus (TEV) protease recognition site for subsequent peptide cleavage release. The plasmid encoding BmK-M9 then was transformed into the *E. coli* strain BL21(DE3) for recombinant toxin production.

Bacteria were grown in LB broth at 37°C with shaking at 180 rpm. BmK-M9 expression was induced with 500 μM IPTG at an OD600 of 0.8, and the cells were grown at 16°C with shaking at 100 rpm for a further 12 h before harvesting by centrifugation for 10 minutes at 10,000-fold gravitational acceleration. The fusion protein was extracted from the bacteria by cell ultrasonication and then captured by passing the extract (buffered in 20 mM Tris, 0.5 mM NaCl, pH = 8.0) over Ni-NTA resin (Qiagen 30230). Nonspecifically-bound proteins were removed by washing with 20 mM imidazole. The fusion protein was eluted with 500 mM imidazole. The eluted fusion protein was lyophilized and further purified by FPLC (Resource S GE 6 mL) to remove imidazole and obtain higher purity recombinant fusion protein.

Added to 1 mg of the fusion protein was 10 U TEV protease, and then the cleavage reaction was allowed to proceed at 16°C for 12 h at a constant volume with TEV Protease buffer (50 mM NaH_2_PO_4_, 150 mM NaCl). The sample then was centrifuged at 12,000 rpm, and the supernatant was subjected to further purification using RP-HPLC (C_8_ XBridge OBD). Containing a nonnative N-terminal glycine residue, rBmK-M9 is one residue longer than native BmK-M9.

Given that rBmK-M9 with His-tag also works on sodium channels, we used a His-tag–specific dye, Invision (Invitrogen LC6030), to construct Fluorescent BmK-M9 (F-BmK-M9). Incubated with Invision for 24 h, rBmK-M9 was subjected to RP-HPLC purification. The single peak was collected and lyophilized for further imaging experiments.

### F-BmK-M9 imaging

HEK293T cells transfected with sodium channels (rNa_V_1.4 and fNa_V_1.4) were incubated with F-BmK-M9 for 1 h in 2 mM Ca^2+^ Ringer's solution (140 mM NaCl, 5 mM KCl, 2 mM MgCl_2_, 10 mM Glucose, 2 mM CaCl_2_, and 10 mM HEPES, pH = 7.4) before fluorescence imaging recordings. Fluorescence images of HEK293T cells incubated with F-BmK-M9 were acquired by using an Olympus IX-71 microscope with a Hamamatsu R2 camera controlled by MetaMorph software. F-BmK-M9 was excited by a LED light source (X-Cite 120LED, Lumen Dynamics) with a 560 nm excitation filter, while fluorescence emission was detected by a 590 nm emission filter.

### Insecticidal assays

Dissolved in insect saline, 10 μM rBmK-M9 were injected into the abdomen region of adult American cockroaches (*Periplaneta americana*) and adult German cockroaches (*Blattella germanica*). Insect saline was used as control. Injections were made using a 1.0 mL syringe (B-D Ultra-Fine). A maximum volume of 1.5 mL was injected per *B. germanica* and 3.0 mL for *P. americana*. Thereafter, cockroaches were housed in closed 1 L conical flasks and provided with dry food and water. The lethal effects were then determined after a period of 24 h. For each acute toxicity assay, up to five doses of rBmK-M9 were injected (n = 5 insects per dose). The assay was repeated three times.

### Mutagenesis of toxin and sodium channels

Chimeras rNa_V_1.4 and fNa_V_1.4 used in this study were generated by the overlapping extension method by using In-Fusion HD Cloning Kits and following the user manual (Clontech); all chimeras were verified by DNA sequencing [51]. Each sodium channel point mutation was constructed by using the QuikChange Lightning Site-Directed Mutagenesis Kit (Agilent) and following the instruction manual; all point mutations were confirmed by DNA sequencing.

For each toxin mutant, site-directed mutagenesis was performed by PCR on expression plasmid first and then the corresponding peptide was expressed as described before. Final confirmation of toxin mutants was carried by CD spectra.

### Cut-open VCF and TEVC recordings

Cut-open voltage-clamp fluorometry (VCF) was used to record ionic currents and fluorescence from oocytes [52,53]. *Xenopus laevis* oocyte preparation and cRNA injection was performed as described previously [54]. Briefly, the cRNA of BgNav1 was coinjected into oocytes with that of TipE at a 2:1 molar ratio (50 ng per cell total) for robust expression. Injected oocytes were incubated individually at 18°C for 5 d in ND-96 solution with 1% penicillin-streptomycin at a pH of 7.4. The temperature of three chambers was maintained at 19°C with a controller (HCC-100A; Dagan Corporation). The internal solution contained 113 mM NMG-Mes, 2 mM Na-Mes, 20 mM HEPES, and 2 mM EGTA, pH = 7.4. The external solution contained 95 mM NMG-Mes, 20 mM Na-Mes, 20 mM HEPES, and 2 mM Ca-Mes_2_, pH = 7.4. The glass pipettes were filled with filtered 3 M KCl in 0.5% agarose with a resistance of 0.5 to 1.0 MΩ. For fluorescence measurement experiments, oocytes were labeled with 20 μM methanethiosulfonate-carboxytetramethylrhodamine in a depolarizing solution (110 mM KCl, 1.5 mM MgCl_2_, 0.8 mM CaCl_2_, and 10 mM HEPES, pH = 7.4) on ice for 40 min. Methanethiosulfonate-carboxytetramethylrhodamine was excited by a LED light source (Luminus, PT-121), while fluorescence emission was detected by a 40 × water-immersion objective with a numerical aperture of 0.8 (CFI Plan Fluor; Nikon). Gating currents were recorded with 1 μM TTX in the external solution.

### Cell culture, transient transfection, and electrophysiology

HEK293T cells were cultured in Dulbecco's modified Eagle's medium with 10% fetal bovine serum, penicillin (100 U/ml) and streptomycin (100 mg/ml) at 37°C with 5% CO2. Cells were plated on cover glasses before transfection. Transient transfection was conducted by using Lipofectamine 2000 (Invitrogen) and following the instruction manual.

Electrophysiological experiments were performed between 24–48 h after transfections as previously described [8]. The macroscopic currents were recorded by using a HEKA EPC10 amplifier with the PatchMaster software (HEKA). The borosilicate glass pipettes were pulled and fire-polished to a resistance of 3–4 MΩ. All recordings were performed at room temperature. To evoke sodium channel currents, a holding potential of −80 mV was used with a testing pulse to −10 mV. The association and dissociation traces were determined using a rapid solution changer (RSC-200, BioLogic) to deliver different concentrations of BmK-M9 and toxin mutants. The stable current amplitude before and after BmK-M9 application was recorded. For the sodium channels recording, the standard pipette solution contained 140 mM CsF, 1 mM EGTA, 10 mM NaCl, 3 mM KCl, and 10 mM MgCl_2_, pH = 7.3. The standard bath solution was 140 mM NaCl, 3 mM KCl, 1 mM MgCl_2_, 1 mM CaCl_2_, and 10 mM HEPES, pH = 7.3.

### Lipid membrane interaction

HEK293T cells were incubated with 10 μM rBmK-M9 for 24 h. The incubated cells were then resuspended in 2 mL of PBS and then centrifuged at 18,000-fold gravitational acceleration for 1 h at 4°C. The supernatant was filtered and subjected to RP-HPLC detection.

Given that rBmK-M9 with His-tag also works on sodium channels, we used a His-tag specific antibody (CST) to prove the interaction between BmK-M9 and lipid membranes. Cells of HEK293T transfected with rNa_V_1.4 and liposome were incubated with 10 μM rBmK-M9 for 24 h and then resuspended in 2 mL of PBS and centrifuged at 18,000-fold gravitational acceleration for 1 h at 4°C. The two kinds of cell pellets were washed three times with PBS to get rid of the residual free toxins, then lysed with RIPA and centrifuged at 18,000-fold gravitational acceleration for 1 h at 4°C. The two kinds of supernatants were collected and detected by western blot for the presence of the toxin.

### Construction of BmK-M9 and rNa_V_1.4 channel model

The structure of BmK-M9 was predicted by backrub protocol using the Rosetta molecular modeling suite version 2016.20. A partial model of rNa_V_1.4 was constructed from L250 to E1600 by membrane-symmetry-loop modeling using the Rosetta molecular modeling suite version 2016.20. The cryo-EM structure of EeNa_V_1.4 (5XSY) was used as the template, the S3–S4 linker and the S4–S5 linker were modeled de novo with the KIC loop modeling protocol. Each round generated 10000 models; among these models, the top 10 lowest-energy models were selected as the inputs for next round of loop modeling. After several rounds of KIC loop modeling, the top 10 models converged well. The lowest energy model was finally selected as the rNa_V_1.4 model.

### Docking of BmK-M9/rNa_V_1.4 complexes

RosettaDock application from Rosetta program suite version 3.4 was used to dock BmK-M9 to rNa_V_1.4 models. Models of the transmembrane domains of rNa_V_1.4 were first relaxed in a membrane environment using the Rosetta-Membrane application. BmK-M9 was initially placed roughly in the center of the binding pocket defined by S3, S3–S4 linker and S4 segments. From the results of double mutation cycle experiments, the distances between D1428-K62 and K1432-Y42 were constrained to move within a 4 Å diameter sphere. After docking, the top 1,000 models with the lowest total energy score were first selected. They further were scored with the binding energy between the ligand and the channel. The top 10 models with the lowest binding energy were identified as the candidates. The model with the lowest binding energy among the largest cluster of the top 10 models was used as the representative model.

### Data analysis

Offline data analysis was performed using IgorPro (WaveMetrics) as previously reported [55]. Voltage–activation relationships were obtained by measuring currents elicited by step depolarizations of 10 mV from a holding potential of −100 mV and calculating peak conductance (G_Na_) using the following equation: *G = I_Na_/(V_m_—E_rev_)* where G is peak conductance, I_Na_ is peak inward sodium current, V_m_ is the test potential and E_rev_ is the reversal potential. The normalized conductance was fitted to a two-state Boltzmann function: *G/G_max_ = [1 + exp(V—V_1/2_)/k]^−1^*, where V_m_ is the voltage potential of the pulse, V_1/2_ is the voltage at half-maximal activation, and k is the slope factor. The voltage dependence steady-state inactivation was determined using 200 ms inactivating prepulses from a holding potential of −120 to 40 mV in 10 mV increments and followed by test pulses to −10 mV for 50 ms. The peak current amplitude during each test pulse was normalized to the maximum current amplitude. The steady-state inactivation data were fitted using a Boltzmann equation: *I/Imax = [1 + (exp(V—V_1/2_)/k)]^−1^*, where V_1/2_, V and k represented the voltage at half-maximal activation, test potential and slope factor, respectively. Dose–response curves to determine LD50 values were fitted using the following form of the logistic equation: *y = 1/(1 + [x]/Dose50)^nH^*, where x is the toxin dose and nH is the Hill coefficient. Nonlinear curve-fitting of data were performed using IgorPro.

## Supplementary Material

nwz097_Supplemental_Files
