# Supplementary material for: Molecular game theory for a toxin-dominant food chain model
Source: Natl Sci Rev. 2019 Jul 19;6(6):1191–200. doi: 10.1093/nsr/nwz097 (PMC8291550; doi:10.1093/nsr/nwz097)
Supplement: nwz097_Supplemental_Files [file nwz097_supplemental_files.zip › Supplementary_data_Revised.docx]

**Supplementary data**

**Molecular game theory for a toxin-dominant food chain model**

Bowen Li, Jonathan R. Silva, Xiancui Lu, Lei Luo, Yunfei Wang, Lizhen Xu, Aerziguli Aierken, Zhanserik Shynykul, Peter Muiruri Kamau, Anna Luo, Jian Yang, Deyuan Su, Fan Yang, Jianmin Cui, Shilong Yang and Ren Lai

**
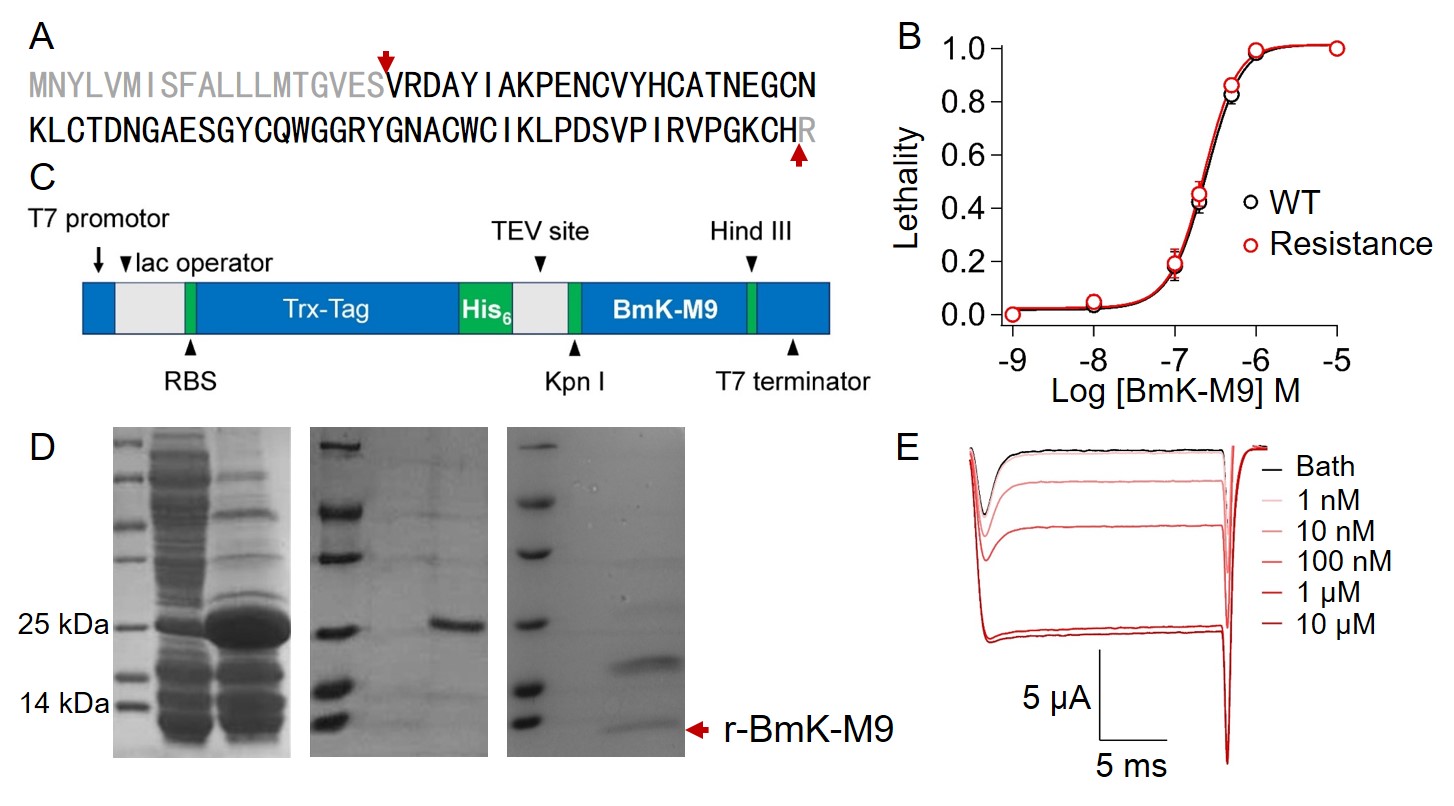
**

**Supplementary Figure 1.** **BmK-M9 is a potent modulator of sodium channels.** (A) The amino acid sequence of BmK-M9 whereby the mature chain is pointed by red arrows. (B) A dose-response curve for the lethal effect of BmK-M9 determined 24 hours after feeding to two kinds of mosquito larvae (wild type *Anopheles sinensis* and Pyrethroid resistant *Anopheles sinensis*). Data points were fitted according to a Hill equation. Average values are given as mean ± SEM. n = 20 mosquito larvae per group. n = 3 groups per data point. (C) Schematic representation of the modified pet32a (+) vector used for prokaryotic expression of BmK-M9. (D) SDS-PAGE gels illustrating different steps in the purification of r-BmK-M9. Fusion protein sample after TEV protease cleavage is pointed by a red arrow (r-BmK-M9). (E) Representative BgNa_V_1 currents from the same oocyte recorded in the presence of five different concentrations of r-BmK-M9.


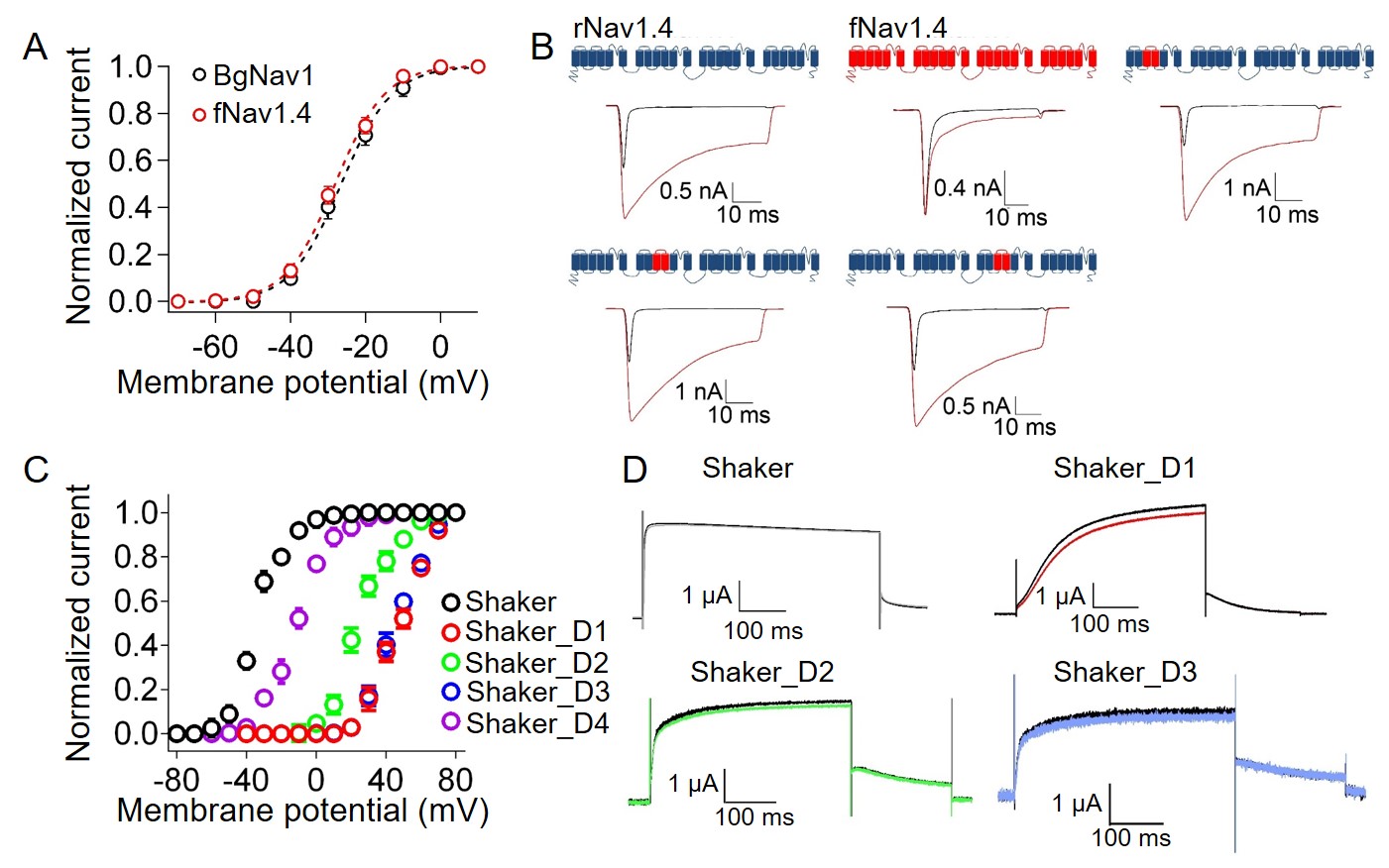


**Supplementary Figure 2. The BmK-M9 sensitivity to chimeric channels.** (A) The voltage-dependent activation traces of fNa_V_1.4 and rNa_V_1.4. The statistical values are given as mean ± SEM (n = 3 cells). Data points are fitted by the Boltzmann equation. (B) Schematic representation of the chimeras between rNa_V_1.4 (blue) and fNa_V_1.4 (red). Representative whole-cell currents of chimeric channels before and after 10 μM BmK-M9 application. (C) Comparison of the conductance-voltage relationship of WT Shaker and the chimeric channels. The statistical values are given as mean ± SEM (n = 3 cells). (D) Representative whole-cell currents recorded before and after 10 μM BmK-M9 application. Chimeras constructed between domain I, II, III VSD paddles of BgNa_V_1 and Shaker (Shaker_D1, Shaker_D2 and Shaker_D3) were expressed in oocytes.


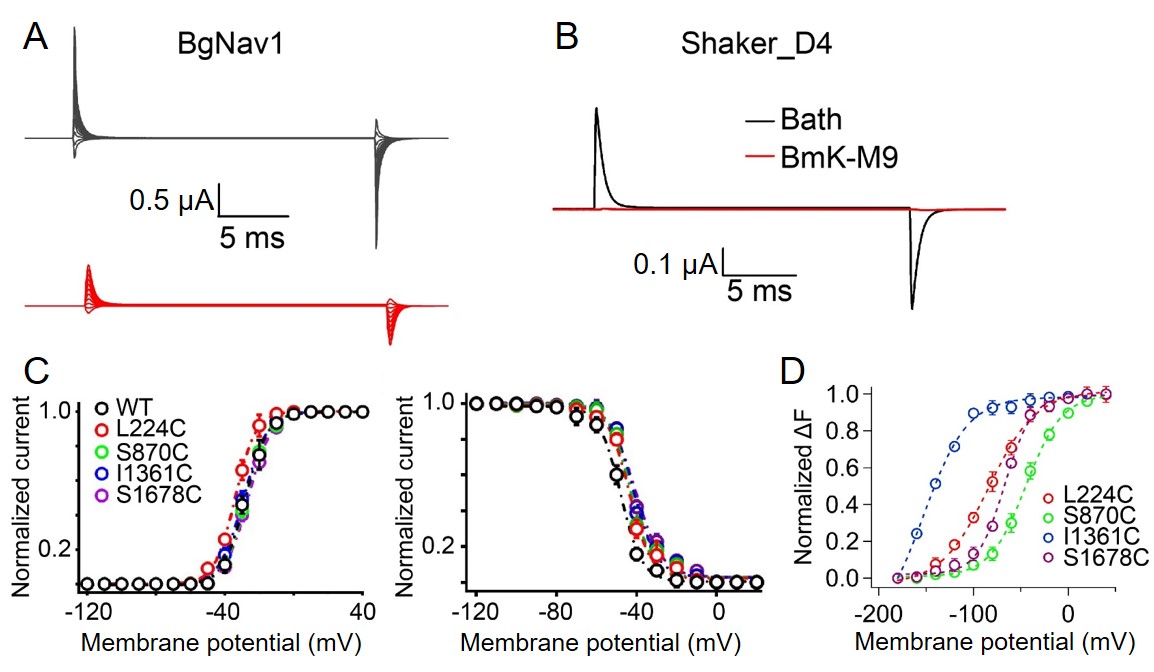


**Supplementary Figure 3. BmK-M9 occludes the movement of DIV-VSD.** (A) Gating currents recorded from BgNa_V_1 before (top panel) and after (down panel) 10 μM BmK-M9 application. (B) Gating currents recorded from Shaker_D4 before (black) and after (red) 10 μM BmK-M9 application. (C) Comparison of the voltage-dependent activation curves (left) and the steady-state inactivation curves (right) of BgNa_V_1 and fluorescence-labeled channel mutants. The statistical values are given as mean ± SEM (n = 3 cells). (D) The voltage-dependent fluorescence curves of four fluorescence-labeled channel mutants.


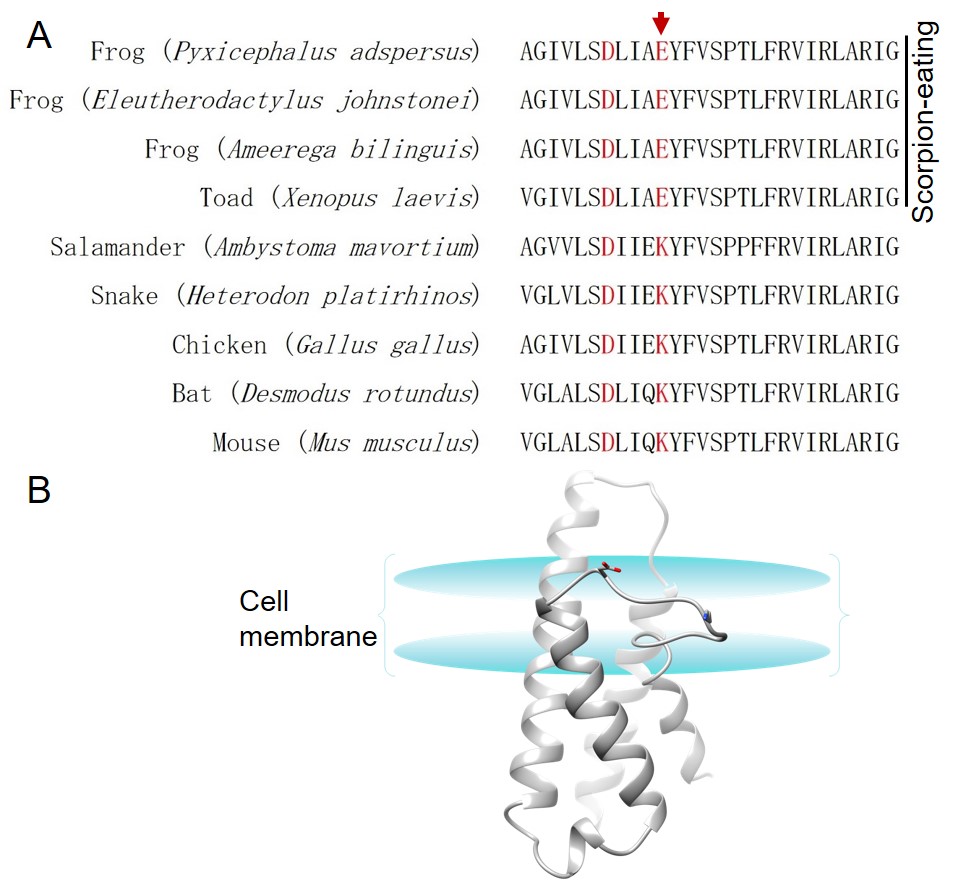


**Supplementary Figure 4. Sequences alignment and the structural model of rNa_V_1.4.** (A) Amino acid sequences alignment of species-specific Asp/Lys-Tyr motif of Na_V_1.4. (B) Resting state model of Asp/Lys-Tyr motif in rNa_V_1.4.

**Supplementary Table 1. Functional comparisons of six scorpion α-toxins**

| Name of  α- toxins | EC_50_ (nM) on BgNa_V_1 | I_5 ms_/I_peak_ on BgNa_V_1 |
| --- | --- | --- |
| BmK-M9 | 136.4 ± 63.1 (n = 3) | 0.992 ± 0.012 (n = 3) |
| BmK-M4 | 225.5 ± 16.5 (n = 3) | 0.904 ± 0.01 (n = 3) |
| BmK-M1 | 150.2 ± 10 (n = 3) | 0.923 ± 0.015 (n = 3) |
| OD2 | 560.3 ± 32.4 (n = 3) | 0.911 ± 0.022 (n = 3) |
| Lqh2 | 1100 ± 70.3 (n = 3) | 0.904 ± 0.026 (n = 3) |
| AaH2 | 1201.4 ± 58.2 (n = 3) | 0.887 ± 0.027 (n = 3) |

**Supplementary Table 2. The I_5 ms_ / I_peak_ values of sodium channels treated with 1 μM BmK-M9.**

| **Subtypes and Species** | **I_5 ms_ / I_peak_** |
| --- | --- |
| Na_V_1.2  Na_V_1.3  Na_V_1.4  Na_V_1.5  Na_V_1.6  Na_V_1.7  fNa_V_1.4  chicken Na_V_1.4  bat Na_V_1.4 | 0.210 ± 0.0021 (n = 3)  0.461 ± 0.0032 (n = 3)  0.877 ± 0.0028 (n = 3)  0.601 ± 0.0057 (n = 3)  0.252 ± 0.0062 (n = 3)  0.177 ± 0.0049 (n = 3)  0.114 ± 0.0071 (n = 3)  0.805 ± 0.017 (n = 3)  0.739 ± 0.013 (n = 3) |

**Supplementary** **Movie 1. Frog directly preys on scorpion without paralytic response.**

**Supplementary** **Movie 2. The interaction between the scorpion and mouse.**
